# Global ethylene oxide uses in foods and considerations for food safety

**DOI:** 10.1057/s41271-026-00659-0

**Published:** 2026-07-24

**Authors:** Laura Chung, Susan Buchanan

**Affiliations:** University of Illinois at Chicago School of Public Health, Chicago, IL USA

**Keywords:** Ethylene oxide, Food safety, Human carcinogen, Food law

## Abstract

Ethylene oxide (EtO) is a sterilant widely used for medical devices, but its use is controversial due to its carcinogenicity. In some countries, EtO is also used to fumigate food to eliminate foodborne pathogens, though this practice is banned in others. National regulations on EtO use in food vary widely; exporting countries prioritize trade facilitation and prevention of foodborne illness, while importing countries emphasize maintaining food safety and promoting domestic public health. These divergent public health priorities present challenges to establishing universal maximum residue limits (MRLs) for EtO and 2-chloroethanol (2-CE) and have resulted in the rejection of food items treated with EtO by some importing national authorities. In response, exporters have advocated for internationally harmonized tolerance levels for EtO and its derivatives to facilitate trade. This paper reviews the use of EtO in food products and the potential health effects, describes conflicting perspectives among global stakeholders, and explores the need for harmonized international standards.

## Key messages

- Ethylene oxide is used as a food sterilant in some countries despite its status as a known carcinogen, while its use is banned in other countries.
- Various countries and international food trade organizations promulgate policies pertaining to the use of ethylene oxide in food that are contradictory.
- Global harmonization of ethylene oxide use in food is lacking, causing challenges to international import/export policy

## Introduction

Since 2020, European authorities have issued a series of food alert notifications and recalls on sesame seeds, spices, herbs, nuts, and instant noodles imported from various countries due to the presence of ethylene oxide (EtO), a known human carcinogen [1]. Several food manufacturers in Europe who used these contaminated ingredients were also required to issue product recalls [2]. Since then, exported spices and food items from many developing countries have been rejected by importing national authorities [2]. Exporters have lost millions in transactions, and food manufacturers in importing nations have also suffered reputational damage for using contaminated ingredients [3]. Discussions around the use of EtO in the food trade are ongoing in the international community, highlighting differing perspectives on its role in food safety and environmental public health [4]. Harmonization of international standards through the Codex Alimentarius, a joint Food and Agriculture Organization/World Health Organization (FAO/WHO) body that sets standards for safe food trade, is expected to clarify the obligations of trading partners and reduce the likelihood that food will be rejected at national borders. However, such harmonization requires consensus among Codex Alimentarius members regarding acceptable standards and has not yet been accomplished. This paper explores the conflicting perspectives among various actors in the global food trade pertaining to the use of EtO and the value of harmonized international EtO standards related to its use in food. We performed a review of the recent literature to synthesize the current state of international law and policy governing the regulation of ethylene oxide in the global food trade and to enhance public understanding of food safety within the context of an increasingly globalized food supply chain.

## Data and methods

Given the limited body of peer-reviewed scholarship on this topic, this review draws extensively from gray literature, including reports from news media, official government publications, materials issued by international organizations, statutory instruments, and decisions and opinions of the European Commission. Information on health risk of exposure to ethylene oxide was taken from searches of PubMed.

## Food safety standards

Food safety is the assurance that food is fit for human consumption and not harmful to health [5]. The modern food safety concept encompasses holding business owners responsible for ensuring safety and mandating traceability of the supply chain from “farm to fork.” [5, 6] Due to global trade, this requires food supply channels to be traceable such that all parties can be held accountable [7].

**Fig. 1:**
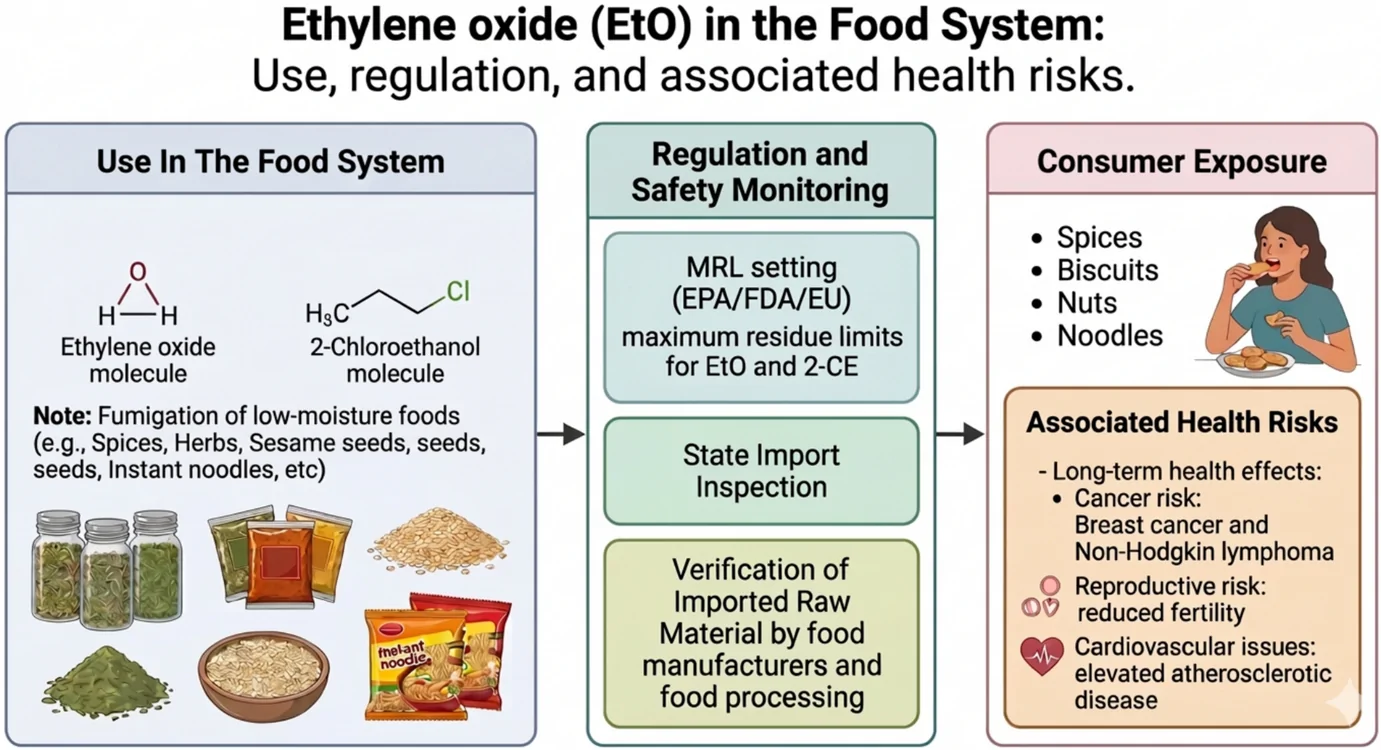
Ethylene oxide in the food system

Figure 1 depicts the use of ethylene oxide in the food system, key regulatory control points throughout the process, consumer exposure pathways, and associated health risks.

## Use of ethylene oxide in food

Ethylene oxide (C₂H₂O; EtO) is a colorless flammable gas with a sweet odor that is used as a sterilizing agent for medical devices, a pesticide for food storage, and a raw material in producing ethylene glycol (antifreeze) and other chemicals [16]. It is an extremely reactive compound with excellent penetrability and antimicrobial properties [16, 17]. For medical device sterilization, EtO is particularly useful for its ability to penetrate polymers and heat-sensitive materials without causing damage [17, 18]. It is estimated that more than 50% of the medical devices now produced in the U.S. are sterilized by EtO [19].

Given its rapid dissipation and excellent sterilizing abilities preventing microorganism growth in treated foods for an extended period, EtO has been used to sterilize spices, sesame seeds, nuts, and other dry foods in some countries, including India, Canada, and the United States (U.S.) [2, 20] The primary mechanism of food sterilization is the fumigation of dry food products with EtO gas in an enclosed space, whereby EtO gas penetrates food materials to alkylate cellular constituents—including proteins, DNA, and RNA—thereby inactivating pathogens [21]. EtO is reported to be nearly ten times more effective than other fumigants such as methyl bromide and phosphine [17]. The use of EtO as a sterilizing agent in recent years has resulted in significant reductions in imported foods being rejected because of contamination with foodborne pathogens [2]. The pressure to assure sterilized food products was driven in part by the opportunity for global food trade [22, 23]. Prior to using EtO, food items such as spices, sesame seeds, and nuts from developing countries frequently faced importation rejections due to contamination with salmonella and other foodborne illness pathogens [24]. EtO fumigation is effective in reducing pathogen levels below detection in a matter of minutes to hours [25].

Despite its usefulness, the use of EtO has been contentious in countries such as the U.S. and the European Union (EU) because it is known to be a potent human carcinogen. The International Agency for Research on Cancer (IARC) has classified EtO as a Group 1 carcinogen (carcinogenic to humans) [11]. EtO is mutagenic, genotoxic, and carcinogenic and has been shown to induce chromosomal damage in humans [14]. Low-dose exposure to ethylene oxide emissions has been linked to increased incidences of breast cancer, non-Hodgkin lymphoma, multiple myeloma, and lymphocytic leukemia among workers in medical device sterilization facilities and even residents living near such facilities [9, 11, 14]. Strict environmental exposure limits have been set by the U.S. Environmental Protection Agency (U.S. EPA) for air levels in the workplace and emissions from factories and sterilization facilities due to evidence that low-level, chronic respiratory exposure increases cancer rates in workers and nearby residents [23]. While human data on oral toxicity of ethylene oxide are limited [8], a 1982 study by Dunkelberg demonstrated that rats administered ethylene oxide intragastrically in oil over a 3-year period developed squamous cell carcinomas in a dose-dependent manner [26]. This finding is consistent with expectations based on physiologically based pharmacokinetic modeling and the molecular properties of ethylene oxide, including its small size, ready gastrointestinal absorption, and ability to form covalent adducts with proteins and nucleic acids, resulting in genotoxic and carcinogenic effects [8]. Table 1 displays the summary evidence of health effects from exposure to EtO. The references represent the current peer-reviewed literature pertaining to human health effects, as noted in the referenced studies and US national agency publications.

**Table 1:** Ethylene oxide health risks

Category	Specific risk/marker	Evidence and observations
Carcinogenicity	Breast cancer	Increased incidence observed among female workers with long-term occupational exposure [8, 9].
	Lymphoid cancers	Links EtO to non-Hodgkin lymphoma and multiple myeloma in sterilization workers [10, 11].
	Leukemia	Lymphocytic leukemia observed in workers with high cumulative exposure [11, 12].
	Stomach and pancreatic cancer	Suggested increases in mortality, though evidence is less consistent than for lymphoid and breast cancers [13].
Genotoxicity/DNA damage	DNA adduct formation	EtO directly alkylates DNA, forming N7-(2-hydroxyethyl)guanine, a marker of mutagenic potential [11, 14].
Reproductive effects	Spontaneous abortion & reduced fertility	Increased risk among exposed female workers in sterilization facilities [8, 15].
Neurological effects	Peripheral neuropathy	Chronic high-level exposure is associated with numbness, weakness, and impaired nerve conduction [8].
Respiratory effects	Irritation & pulmonary toxicity	Acute high-level exposure can cause respiratory irritation, pulmonary edema, and lung injury [8].
Dermal/direct exposure	Eye and skin irritation	Direct irritant effects at high concentrations; can cause chemical burns and blisters [13].

When EtO is used to sterilize food products, it is generally assumed that most of it will dissipate before consumption. Unfortunately, some of the EtO lingers, especially when ventilation is inadequate [4]. Dudkiewicz et al. (2022) suggested that food chemistry rapidly changes when heat is applied to EtO-contaminated ingredients during manufacturing processes, causing most residual EtO to evaporate or react with water to form ethylene glycol, a safer compound, potentially rendering recalls of all food products manufactured with fumigated ingredients unnecessary in the EU [3]. However, this may not apply to all situations, as many of the foods treated with EtO—such as nuts and spices—are often consumed directly from the packaging without further heat processing.

Even though much of the EtO used for fumigation dissipates during ventilation, the residue 2-chloroethanol (2-CE) forms when EtO reacts with chlorides naturally found in food. Since 2-CE is a more stable compound and does not easily dissipate, it often remains in detectable concentrations [4]. As an industrial chemical, 2-CE is known to be acutely toxic if inhaled. The carcinogenic effects of 2-CE are believed to be less than those of EtO based on outdated studies, but these studies are inadequate to meet modern food science standards to measure its degree of carcinogenicity and mutagenicity or to determine its safe levels in food commodities [4, 26, 27]. Regardless of whether the potential health effects of ingestion of 2-CE residue in EtO-treated foods is known, its presence functions as a marker for former fumigation with EtO [26, 28].

## Ethylene oxide maximum residual levels

Maximum residual levels (MRLs) are the maximum amount of pesticide or processing agent residues legally permitted in food or animal feed [29]. MRLs are also referred to as food tolerances in the U.S. Typically, national food safety or environmental health agencies set MRLs for pesticides and processing agents based on their safety data for human health. In the U.S., while the U.S. Food and Drug Administration (U.S. FDA) sets tolerance levels for food additives, the MRLs for EtO are set by the U.S. EPA because EtO is considered a “pesticide” or a “processing aid,” not a food additive or ingredient [23, 30]. In the U.S., a residual level of EtO is permissible for specific food commodities, including spices, dry herbs, and nuts, under the Federal Insecticide, Fungicide, and Rodenticide Act (FIFRA) 21 CFR §101.100(a)(3), and those who use EtO within the U.S. must obtain a permit and follow stringent requirements about how and when it is used [31]. In January 2025, the U.S. EPA issued a final interim decision on MRLs of EtO, which are currently set at 7 ppm for herbs, spices, and sesame seeds, and 50 ppm for walnuts. The MRLs for the EtO derivative, 2-CE level, are set at 940 ppm for these same food items [23].

Several other countries have promulgated MRLs for EtO in food or banned its use in food altogether (Table 2) [32]. In particular, the EU has banned the use of EtO for fumigation of food products and has set very strict MRLs for imported foods. The EU position is that EtO is category 1B carcinogen and a mutagen, and no safe level for human consumption can be set for a carcinogenic substance [33]. In such cases, the MRL will be set at the level of detection. The EU also maintains a robust importation food inspection system, enabling authorities to identify violations [2]. In the EU, MRLs for EtO are calculated by combining the concentrations of EtO and 2-CE using the formula: sum EtO and 2-CE = conc EtO + (conc 2-CE × 0.55) [28]. According to EC Regulation 396/2005, the MRLs for sum EtO and 2-CE in food are to be set at the limit of quantification, which are currently enforced at 0.01 to 0.1 ppm based on the type of commodity [4]. While the contamination levels with EtO and its derivatives can vary widely, according to the Office for Risk Assessment and Research in the Netherlands, contamination levels of EtO and 2-CE combined in imported sesame seeds commonly range from 1 to 10 ppm [26].

**Table 2:** Maximum residual levels (MRL) of ethylene oxide

Country or economic region	Ethylene oxide (EtO) MRL	Foods included	Notes
European Union	0.01 to 0.1 ppm for the sum of EtO and 2-CE	All food commodities	Sum of EtO and 2-CE = conc EtO + (conc 2-CE × 0.55).[28].
United States	7 ppm EtO	Spices, herbs, some nuts	The U.S. EPA publishes separate MRLs for EtO and 2-CE [23].
	50 ppm EtO	Walnuts	
	940 ppm 2-CE	Various commodities	
Canada	7 ppm EtO	Spices, dried herbs, sesame seeds	No MRL exists for walnuts in Canada [36].
	940 ppm 2-CE	Various commodities	
New Zealand	0.1 mg/kg EtO [19, 20]	All foods	
Australia	N/A	N/A	EtO fumigation of food banned since 2003 [34].
Hong Kong	N/A	All foods	Food with any detectable level of EtO is not permitted [4].
Taiwan	N/A	N/A	“EtO and its metabolite 2-CE shall not be detected in food.” [4]
Thailand	N/A	N/A	EtO is classified as Type 4 hazardous substance, and food including EtO is prohibited [4].
Singapore	50 ppm EtO	Whole spices only	Any other food containing detectable residue of EtO is not permitted [4].
Indonesia	N/A	N/A	EtO use as a pesticide is prohibited [4].
South Korea	0.01 ppm EtO	All food commodities [4]	
Japan	0.01 ppm EtO	All food commodities [4]	

*ppm* parts per million, *MRL* maximum residue limits, *2-CE* 2-chloroethanol

As shown in Table 2, MRLs for EtO vary widely. The U.S. and Canada allow up to 7 ppm of EtO for several food items and up to 940 ppm of 2-CE for these food items. In fact, the U.S. permits up to 50 ppm of EtO and 940 ppm of 2-CE specifically for walnuts. By contrast, the EU sets MRLs that may fall below the limit of detection, as the EU combines EtO and 2-CE concentrations into a single metric: “sum EtO and 2-CE.” Because the 2-CE levels are often one to two orders of magnitude higher than residual EtO after food fumigation, the inclusion of 2-CE enables the EU to reject foods even when EtO is undetectable [28]. For example, a batch of sesame seeds containing no detectable EtO and 224.7 μg/kg of 2-CE, as measured by gas chromatography, would be deemed to have a “sum EtO and 2-CE” of 0.1229 ppm [sum EtO and 2-CE = conc EtO + (conc 2-CE × 0.55)] [28]. This batch would be rejected under the EU’s MRL of 0.05 ppm for sesame seeds, but would comply with the U.S. MRLs of 7 ppm for EtO and 940 ppm for 2-CE. Several countries, including Australia, New Zealand, and Hong Kong, do not permit any detectable level of EtO in food products [32, 34, 35].

The wide range and differing specifications related to the national maximum residual levels shown in Table 2 illustrate the challenges to enforcement and compliance at the global level. The consequences of the lack of global harmonization related to EtO used in food sterilization are likely seen in divergent public health outcomes as well as challenges to international food trade.

## International law on food safety

No unified international legal framework exists on food safety. Instead, a patchwork of several international agreements and guidelines exists under international organizations such as the World Health Organization (WHO), the Food and Agricultural Organization (FAO), and the World Trade Organization (WTO) [37, 38]. In addition, each nation has its own national laws that regulate trade and perform food safety inspections at the border, forming a complex web of regulations that differ in each country.

The WTO promotes free trade and provides “most-favored-nation” status to all member nations, discouraging discrimination among trading partners, such as reciprocal lower customs duty rates for a particular nation or specific goods [39]. The WTO now boasts 166 member countries and has opened an era in which farmers from developing countries can access markets in industrialized nations through reduced trade barriers [40]. However, by reducing these barriers, the WTO and specifically its Agreement on Agriculture can impinge on national authorities’ ability to regulate the importation of food from other member countries that have lower food safety standards [41]. The WTO Agreement on Agriculture came into force in the U.S. in 1995, when the U.S. became a member country of the WTO [29].

The Codex Alimentarius Commission is a joint FAO/WHO body that sets standards for safe food trade [42]. It was established in 1963 to develop international food standards that ensure consumer health and fair practices in international food trade. It currently boasts 189 member countries and regions [43]. No Codex Alimentarius standards currently exist for EtO MRLs in food [32].

## National laws on ethylene oxide

Two important regions with laws regulating EtO use in food are the U.S. and Europe, major importers of global spices. The U.S. spice and seasoning market size in 2025 is estimated at 2.94 billion U.S. Dollars (USD), making the U.S. the nation with the single largest spice importation [44]. About 80 to 90% of spices approved by the American Spice Association of America are treated spices from India [45, 46]. The European spice and seasoning market in 2025 is estimated at 6.5 billion USD [47], and various Asian-Pacific countries, including China and India, combined make up a market size of approximately 5.58 billion USD [48].

### U.S. food regulations on ethylene oxide

U.S. regulations concerning EtO are complex [49]. The U.S. FDA enforces the MRLs set by the U.S. EPA by conducting inspections of both domestic and imported foods [23, 30, 50]. Meanwhile, the U.S. Department of Agriculture (USDA) collaborates with the U.S. EPA to evaluate safe agricultural practices and oversees the certification process for the “organic” label [51–53]. Because EtO is considered a pesticide, manufacturers and importers are not required to list it as a food ingredient on product labels [30]. However, to bear the USDA “organic” label, food must be produced without synthetic pesticides, fertilizers, genetically modified organisms (GMOs), antibiotics, or irradiation, and must be certified by a USDA-accredited certifying agent [52, 54]. Therefore, food treated with EtO cannot be labeled as organic [52]. Falsely labeling products as “organic” carries both civil and criminal penalties. Under the National Organic Program (NOP) regulations (7 CFR Part 5), civil penalties of up to $18,743 fine per violation may be imposed, adjusted for inflation [55]. Willful violations are also subjected to criminal penalties under the Organic Foods Production Act of 1990, including fines and imprisonment [52].

As noted above, the U.S. permits relatively high MRLs of 7 ppm EtO for various spices, herbs, sesame seeds and nuts, with a higher limit of 50 ppm for walnuts, and separate MRLs of 940 ppm 2-CE in these commodities [23]. In its 2025 Interim Decision, the U.S. EPA acknowledged that human safety data regarding EtO and 2-CE in food remain incomplete and has requested that future registrants submit additional safety data—an effort that may pose challenges for new applicants due to the associated research costs [23].

The U.S. MRLs make the U.S. one of the most permissive industrialized nations in terms of EtO residue tolerances. These high tolerance levels reflect the regulatory model under which U.S. federal agencies operate, in which any restriction on business interests must be based on scientific evidence. According to the Foundations for Evidence-Based Policymaking Act of 2018 (Public Law 115–435), federal agencies such as the FDA and EPA are required to use evidence to support their policymaking decisions [56]. Evidence may include scientific data, statistical analysis, or epidemiologic data [56, 57]. In essence, the burden of providing evidence lies with the government to demonstrate a lack of safety in order to impose regulations that restrict commercial practices.

### European food regulations on ethylene oxide

The EU is a political and economic union of 27 member nations that allows the free movement of goods and food among its members [2, 4, 58, 59]. The use of EtO in the fumigation of food and animal feed has been banned since 2011 [60]. If contamination is detected above the EU MRL, a recall notice must be issued for the affected food product [60]. The use of EtO is still allowed for the sterilization of medical devices however [60].

According to Kowalska & Manning (2022), the EU issued 262 notifications related to EtO violations in imported food via the EU Rapid Alert System for Food and Feed (RASFF) in 2020 [2]. By February 2025, more than 900 EtO-related notifications had been issued for food originating from several countries, including India, China, Ukraine, Pakistan, Turkey, Lebanon, Indonesia, Morocco, Vietnam, and Hong Kong [4, 61]. Five years earlier, Indian sesame seeds received 65 RASFF notifications due to salmonella contamination. Exporters may have been fumigating sesame seeds with EtO to suppress salmonella contamination, a practice prohibited in the EU [59]. Some sesame seeds were found to contain “sum EtO and 2-CE” levels as high as 186 mg/kg, which is approximately 3,700 times the European maximum residue limit of 0.05 mg/kg for sesame seeds [2]. Processed foods manufactured within Europe that used these adulterated sesame seeds were also subjected to recall [2].

By combining 2-CE concentrations to EtO concentrations, the EU can classify foods with undetectable EtO as contaminated based on the presence of 2-CE. The strict regulatory approach is rooted in the precautionary principle, which the EU applies in the context of environmental and food safety regulations [62]. According to European Commission Regulation No. 178/2002, “in those specific circumstances where a risk to life or health exists but scientific uncertainty persists, the precautionary principle provides a mechanism for determining risk management measures or other actions in order to ensure the high level of health protection chosen in the Community.” [62] Under this principle, when a product is suspected of posing a risk to human health, the EU may take protective measures even in the absence of full scientific certainty. It is the responsibility of the business applicants seeking approval to introduce a product or process to demonstrate its safety through scientific evidence [33].

In terms of pesticide use, similar to the U.S., food treated with EtO cannot bear the EU organic logo. The European Commission has established a uniform organic food labeling system that applies across all EU member nations [63]. The EU organic logo certifies that the product contains no less than 95% organic ingredients, with limited use of artificial fertilizers, herbicides and pesticides, hormones, antibiotics, and ionizing radiation, and that GMOs are not used [64]. Penalties for falsely labeling food as “organic” are determined by individual member nations [65, 66]. Under the EU-U.S. organic equivalence cooperative established in 2012, the EU and U.S. have also agreed to recognize each other’s organic certifications as equivalent [67]. This arrangement is set to expire in 2027 unless extended or renegotiated.

### Asian food regulations on ethylene oxide

India’s annual export of spices during 2023–24 stood at 4.25 billion USD, accounting for 12% of the global spice trade [68]. According to the Crop Care Federation of India (CCFI), the regulatory framework governing the use of EtO in food in India remains unclear [69]. EtO is not registered as a pesticide under India’s Insecticides Act of 1968, and is instead classified as an “industrial chemical of economic importance” [69]. After the EU flagged several Indian spice shipments for EtO contamination, multiple Indian media outlets called for Indian government authorities to defend the use of EtO, arguing that it is not substantially harmful to health [45, 46, 69, 70]. In response to a series of EU recalls of Indian spice products, the Spices Board of India initiated engagement with the Codex Alimentarius Commission of the joint FAO/WHO to advocate for greater tolerance of EtO and 2-CE residues in spices [68].

Enforcement of food safety laws within India has historically been weak. According to 2022–23 data from the Food Safety and Standards Authority of India (FSSAI), approximately 25% of domestic food products checked by the authority were non-compliant with Indian law, due to safety concerns such as pathogen presence, substandard quality, or misbranding or misleading advertisements [71]. In fact, several Indian products found to be contaminated with EtO in foreign markets were also falsely labeled as “organic” [3]. It remains unclear whether domestic products are being tested for EtO content [71].

In 2024, Hong Kong and Singapore banned several spice products from the popular Indian spice brands MDH and Everest [70]. In response, the Spices Board of India mandated EtO testing for all spice shipments headed to Singapore and Hong Kong [70].

## The need to harmonize ethylene oxide law in global food trade

The lack of a harmonized approach to ethylene oxide in global food trade has been noted by entities on both sides of the issue [4, 32]. From the perspective of exporting nations, concerns have been raised regarding reputational damage as safe food exporters due to product bans and rejections of EtO and 2-CE content, especially in the absence of definitive evidence of carcinogenicity of 2-CE. Similar to the medical device industry, EtO has been deemed too useful as a sterilization agent, with arguments made that it facilitates public health by eliminating harmful bacteria. For example, the Chairman of the Federation of Indian Spice Stakeholders (FISS) stated, “This Ethylene oxide treatment is done only for the health of the people… When we treat them with ethylene oxide, microbiological parameters come under control and their dangerous effects are eliminated.” [45].

At the 2024 Codex Alimentarius Commission meeting, the absence of international standards for EtO and 2-CE was cited as a significant barrier to international trade [4, 32]. The wide variations in MRLs have caused trade disruptions, while the risk profile of 2-CE remains insufficiently investigated [32]. Indonesia pointed out that the EU’s approach of combining EtO and 2-CE concentrations under a single MRL treats 2-CE as equivalent to EtO, despite its unproven carcinogenicity [4, 32] and advocated for separate international MRLs for EtO and 2-CE [32]. Indonesia also requested that EtO and 2-CE be included as priority contaminants for evaluation by the Joint FAO/WHO Expert Committee on Food Additives (JECFA), a scientific expert committee jointly administered by the FAO and WHO [4].

Importing countries have different priorities for maintaining food safety. In the U.S. and Europe, cancer is the second leading cause of death, and foodborne illnesses are not among the top ten causes of death [72, 73]. In contrast, India’s list of top causes of death includes diarrheal diseases, pointing out the continued public health threat posed by foodborne pathogens [74]. In the EU, where standards of living are generally high, consumers may be willing to pay more for organic foods produced without pesticides, and most residents expect that grocery store products be free of both pathogens and harmful chemicals [75]. The government has taken the stance that a potentially carcinogenic substance should not be intentionally added to food [60, 75]. These expectations stem from decades of government regulations prioritizing public health above producer profits.

It may be difficult for exporters from some countries to meet these high standards without substantially improving sanitary practices and adopting alternative sterilization methods [76]. Alternative techniques such as irradiation, thermal processing, ozone treatment, steam treatment, or alternative fumigants, often require substantial capital investment or new equipment, may affect the taste or color of herbs and spices, or may lack sufficient penetrability to achieve desired sterility [18, 76–80]. These methods may be technically out of reach, more costly, or may reduce profit margins of exporters in developing economies [76].

In many industrialized nations, governments subsidize the costs of cancer through social welfare and healthcare programs, creating an incentive to keep populations cancer free [81, 82]. If contaminated imported products negatively impact public health, the lost productivity, reduced quality of life, and medical costs are borne by the importing countries. Thus, these countries have a clear interest in upholding strict food safety standards and protecting their public health standards from imported products that do not meet their domestic safety standards [83].

## Conclusions

Importing nations have a vested interest in maintaining the food safety standards their consumers expect. For the EU, this includes an expectation that food will not be intentionally processed with carcinogens, according to their laws [84]. If labeled as organic, the food should not have been treated by EtO or other pesticides [65]. On the other hand, EtO has been found useful in decreasing the contamination of food with microorganisms, which previously was a recurrent barrier to international food trade. While harmonization of international standards generally promotes trade, EtO’s use in food is likely to remain controversial due to divergent local public health priorities. While there is a call to create a Codex standard for EtO in food, any compromise for less than prohibiting its use does not promote the public health priorities of many importing countries. Importers like the EU cannot be expected to lower their own public health standards or tolerate EtO-treated food when safer food sources are available to their populations. And given the domestic imperative to control foodborne pathogens, exporting governments should assess whether EtO use indeed serves the public health needs of their nationals. Moreover, taking strict measures to prevent false labeling of EtO-treated food as organic in the export context will minimize reputational damage to the national brands as a source of safe food. The issue of food safety vs. health risk from EtO requires further exploration by regulating bodies at both national and international levels.

## Data Availability

No datasets were generated or analysed during the current study.
